# Acute Neurofilament Light Chain Plasma Levels Correlate With Stroke Severity and Clinical Outcome in Ischemic Stroke Patients

**DOI:** 10.3389/fneur.2020.00448

**Published:** 2020-06-11

**Authors:** Helle H. Nielsen, Catarina B. Soares, Sofie S. Høgedal, Jonna S. Madsen, Rikke B. Hansen, Alex A. Christensen, Charlotte Madsen, Bettina H. Clausen, Lars Henrik Frich, Matilda Degn, Christian Sibbersen, Kate L. Lambertsen

**Affiliations:** ^1^Department of Neurology, Odense University Hospital, Odense, Denmark; ^2^Department of Neurobiology Research, Institute of Molecular Medicine, University of Southern Denmark, Odense, Denmark; ^3^BRIDGE - Brain Research – Inter Disciplinary Guided Excellence, Department of Clinical Research, Odense, Denmark; ^4^Department of Biochemistry and Immunology, Lillebaelt Hospital, University Hospital of Southern Denmark, Vejle, Denmark; ^5^Department of Regional Health Research, University of Southern Denmark, Odense, Denmark; ^6^The Orthopaedic Research Unit, Department of Clinical Research, Odense, Denmark; ^7^OPEN, Open Patient data Explorative Network, Odense University Hospital, Department of Clinical Research, University of Southern Denmark, Odense, Denmark; ^8^Pediatric Oncology Laboratory, Department of Pediatrics and Adolescent Medicine, University Hospital Rigshospitalet, Copenhagen, Denmark; ^9^Mental Health Services in the Region of Southern Denmark, Odense, Denmark

**Keywords:** modified rankin scale, Scandinavian Stroke Scale, biomarkers, functional outcome, transient ischemic attack

## Abstract

**Background:** Ischemic stroke causes increased blood–brain barrier permeability and release of markers of axonal damage and inflammation. To investigate diagnostic and prognostic roles of neurofilament light chain (NF-L), we assessed levels of NF-L, S100B, interleukin-6 (IL-6), E-selectin, vascular endothelial growth factor-A (VEGF-A), vascular cell adhesion molecule-1 (VCAM-1), and intercellular adhesion molecule-1 (ICAM-1) in patients with acute ischemic stroke or transient ischemic attack (TIA) and healthy controls.

**Methods:** We studied neurofilament (NF) expression in 2 cases of human postmortem ischemic stroke, representing infarcts aged 3- to >7-days. In a prospective study, we measured plasma NF-L and inflammatory markers <8 h of symptom onset and at 72 h in acute ischemic stroke (*n* = 31), TIA (*n* = 9), and healthy controls (*n* = 29). We assessed whether NF-L, S100B, and IL-6 were associated with clinical severity on admission (Scandinavian Stroke Scale, SSS), diagnosis of ischemic stroke vs. TIA, and functional outcome at 3 months (modified Rankin Scale, mRS).

**Results:** NF expression increased in ischemic neurons and in the infarcted brain parenchyma after stroke. Plasma NF-L levels were higher in stroke patients than in TIA patients and healthy controls, but IL-6 levels were similar. Higher acute NF-L levels were associated with lower SSS scores at admission and higher mRS scores at 3 months. No correlation was observed between NF-L and S100B, NF-L and IL-6, nor between S100B or IL-6 and SSS or mRS. Compared to controls, stroke patients had significantly higher VEGF-A and VCAM-1 at <8 h that remained elevated at 72 h, with significantly higher VEGF-A at <8 h; ICAM-1 was significantly increased at <8 h, while S100B and E-selectin were unchanged.

**Conclusions:** Plasma NF-L levels, but not IL-6 and S100B, were significant predictors of clinical severity on admission and functional outcome at 3 months. Plasma NF-L is a promising biomarker of functional outcome after ischemic stroke.

## Introduction

Ischemic stroke is the second leading cause of death and disability in the Western world^1^ with symptoms ranging from complete remission within 24 h (transient ischemic attack, TIA) to lasting disability in the form of cognitive dysfunction, physical disability, and complete dependency on others. The currently available acute treatments of intravenous administration of tissue plasminogen activator (1, 2) and mechanical thrombectomy (3) are associated with rare but severe side effects such as hemorrhage of the brain and body. Because these treatments require rapid initiation to be effective, some patients may be exposed to side effects without benefiting from the treatment. We need, therefore, to identify biomarkers that can predict functional outcome, especially in the early phases, and improve our understanding of the pathophysiological mechanisms underlying tissue damage following a stroke to develop new advanced therapeutic strategies.

When brain damage occurs, neuronal injury and disruption of axonal membranes lead to the release of cytoskeleton proteins, such as neurofilaments (NFs), into the interstitial fluid and eventually into the cerebrospinal fluid (CSF) and blood. NFs are highly specific structural, neuronal cytoskeletal proteins that consist of four NF subunits: NF light (NF-L), NF medium (NF-M), and NF heavy (NF-H) chains, and alpha-internexin. NF-L has been studied as a potential CSF and circulation biomarker for a wide range of neurological disorders (4–7), including cerebral small vessel disease (8) and subacute ischemic stroke (9, 10).

The inflammatory process that ensues after a stroke destabilizes the blood–brain barrier (BBB) and contributes to neuro-axonal damage, thereby increasing the release of NFs and glial and inflammatory markers into the CSF and blood (11). Ischemic stroke has recently been shown to cause persistent elevations in serum NF-L that correlated with infarct volumes and recurrent ischemic lesions (12). Furthermore, NF-L levels obtained within 24 h of symptom onset in the blood of ischemic stroke and TIA patients were associated with clinical severity on admission and TIA diagnosis, but not with infarct size or functional outcome at 3 months (13). Plasma levels of the pro-inflammatory cytokine interleukin-6 (IL-6) on days 5–7 after symptom onset have previously been suggested to be a reliable biomarker in ischemic stroke and to correlate with brain infarct volume, stroke severity, and long-term outcome (14).

Using the very sensitive single-molecule array (simoa) technique (15), we determined plasma NF-L concentrations in ischemic stroke and TIA patients, and healthy controls and their association with disability scores, functional outcome, S100B, and IL-6 levels. We also investigated changes in plasma levels of E-selectin, vascular endothelial growth factor-A (VEGF-A), vascular cell adhesion molecule-1 (VCAM-1), and intercellular adhesion molecule-1 (ICAM-1) in blood samples obtained <8 h and at 72 h after symptom onset and compared these to levels in healthy controls.

## Materials and Methods

### Participants

Patients were included consecutively from September 2017 to April 2018 and comprised patients admitted to the Department of Neurology, Odense University Hospital (OUH), Denmark. Patients presenting with classical clinical symptoms of stroke were eligible if they were over 18 years of age and were admitted within 48 h of symptom onset. Both men and women were included, just as patients undergoing thrombolysis or thrombectomy were included. Exclusion criteria were presence of a space-occupying lesion, sinus thrombosis, pregnancy, or the inability to write and understand Danish.

Healthy controls were recruited from the above patients' relatives (*n* = 7) and from patients aged over 18 years undergoing surgery for chronic rotator cuff tendon tear (*n* = 22) at the Department of Orthopedic Surgery, OUH. Exclusion criteria were diabetes, autoimmune disease, previous shoulder surgery, shoulder fracture or a dislocated shoulder, and the inability to write and understand Danish. None of the orthopedic patients had a prior history of a stroke. All patients and controls provided written informed consent before study participation. Patient information was gathered from patient journals after consent had been given.

### Study Procedures and Clinical Examinations

A standard non-contrast head computed tomography (CT) scan was taken at admission on all patients suspected of stroke. Magnetic resonance imaging (MRI) was performed on 16 patients for further diagnostic clarification. The scans were assessed by radiologists at OUH for ischemic or hemorrhagic changes in the neural tissue. Patients were defined by clinical symptoms, medical history, and physical examination. Cases where symptoms resolved within 24 h were defined as TIA. Cases with persistent symptoms were classified as stroke. None of the TIA patients underwent MRI, whereas 16 stroke patients underwent MRI. All MRI confirmed acute ischemic lesions corresponding to the clinical symptoms. Patients were treated according to the existing national guidelines.

### Disability Assessment

A clinician assessed stroke severity using the Scandinavian Stroke Scale (SSS), which is based on a physical examination and gives a score of 0–58, where a higher score reflects milder symptoms (16). Candidates for thrombolysis or thrombectomy were furthermore assessed with the National Institute of Stroke Severity Scale (NIHSS) and the modified Rankin Scale (mRS) (17). For generalizability, SSS scores were also converted into NIHSS scores using the formula: Baseline NIHSS = 25.68–0.43^*^SSS (18). The mRS was also used to assess functional outcome 3 months after discharge via a telephone interview. The mRS is a 7-step scale (0–6), where 0 represents no functional disability, higher scores reflect less favorable outcome, and death is graded as 6.

### Other Data Collection

The following data were collected from controls and patients or patient records: age, sex, weight, height, smoking and drinking habits, pre-existing diabetes, and use of anti-inflammatory medication. In ischemic stroke patients, we further collected data on first time of clinical symptoms, symptoms at time of admission, and type and location of the lesion. In orthopedic patients, we further collected data on pre-existing rheumatic disease (rheumatoid factor, anti-nuclear antibodies, mitochondrial antibodies, and cyclic citrullinated peptide antibodies). None of the orthopedic patients displayed any signs of rheumatic disease. Body mass index (BMI) was calculated based on height in centimeters and body weight kilograms (body weight/height^2^). As our two control groups (patients' relatives and orthopedic patients) were comparable in age (*p* = 0.09), sex (*p* = 0.65), BMI (*p* = 0.72), drinking habits (*p* = 0.14), and anti-inflammatory medication (*p* = 1), though not for smoking habits (*p* = 0.02), we combined these two groups into one control group for further analysis.

### Blood Sampling and Storage and Biomarker Analysis

Venous blood samples were obtained in neurological patients at admission (*n* = 40; *n* = 31 for ischemic stroke and *n* = 9 for TIA) and again at 72 h after symptom onset in ischemic stroke patients (*n* = 11). Time to first sample from symptom onset in ischemic stroke and TIA patients was 7.5 h ± 5.6 h (mean ± SD) (ischemic stroke patients: median 350 min (IQR; 155–884 min, range 70–2,795 min) and TIA patients: median 255 min (IQR; 148–917, range 65–1,260). In patients undergoing surgery for rotator cuff tendon tear (*n* = 22), blood was drawn just before to surgery. Blood from controls (*n* = 7) was drawn on the day of admission or 14-days after admission. Blood was collected in 4-mL vacutainers and EDTA tubes. The collected blood was centrifuged at 2,000 g for 10 min, plasma samples aliquoted and kept in a −80°C freezer until analysis. Plasma levels of NF-L in controls, TIA, and ischemic stroke patients were measured using a commercially available NF kit (Quanterix, Lexington, MA, USA) for the single-molecule array immunoassay (Simoa) HD-1 Analyzer (Quanterix). An in-house pool was used as an internal control and included in each assay for evaluating assay performance. Samples were analyzed in duplicates, and coefficient of variation (CV) was <12%. Plasma levels of IL-6, E-selectin, vascular cell adhesion molecule 1 (VCAM-1), intercellular adhesion molecule 1 (ICAM-1), and vascular endothelial growth factor (VEGF) in controls and ischemic stroke patients were measured in plasma samples by an electrochemiluminescence immunoassay, using the MSD™ V-PLEX Vascular Injury Panel 2 (VCAM-1 and ICAM-1), the Human E-Selectin Kit, the V-Plex Human VEGF Kit (Mesoscale, Rockville, USA), and the V-Plex Human IL-6 kit according to the manufacturer's instructions (19). Samples were run in duplex. Prior to measurement, the samples were diluted 2-fold in Diluent 41, and MSD Discovery Workbench software was used for analysis. Samples with CV values above 25% in individual analyses were excluded. Plasma Human S100B was analyzed by ELISA (Merck Millipore, Søborg, Denmark) according to the manufacturer's recommendations on a Tecan Infinite M200; all samples were analyzed in duplicates, and a CV of <20% was accepted. The standard curve was fitted by a 4-parameter logistic equation (Magellan Software, Tecan).

### Immunohistochemistry for Neurofilament

For cellular analysis of NF in the ischemic brain, we used postmortem brain tissue from two patients that died from a stroke. One patient was a 59-year-old man with a >7-day-old infarct in the right parietal lobe, and the other patient was a 74-year-old female with a 3–7-day-old infarct in the left temporal lobe. Parallel tissue sections have been part of previous studies on surfactant protein-D, IL-1, and TNF (20–23). Tissue blocks containing the infarcted brain tissue were embedded in paraffin, and 2-μm-thick sections were cut on a microtome. Immunohistochemical staining was performed as routinely done at the Department of Pathology, OUH, using a fully automated VENTANA BenchMark ULTRA immunostainer (Ventana Medical Systems, Tucson, AZ, USA). The immunoreactive product was visualized using the OptiView DAB detection kit (Ventana Medical Systems), and the signal was enhanced with the Ventana Amplification Kit (Ventana Medical Systems). The sections were heated to 75°C for 4 min, deparaffinized at 72°C, and demasked using a T-EG-based buffer (10 mM Tris + 0.5 mM EGTA, pH 9.0). Sections were blocked for endogen peroxidases using 1.5% H_2_O_2_ in Tris-buffered saline (TBS) and incubated with monoclonal mouse anti-NF (phosphorylated and non-phosphorylated NF-H chain) antibody (clone N52, 1:1,000, Sigma-Aldrich) for 1 h. Sections were counterstained with Hematoxylin II (Ventana Medical Systems) using the BenchMark Ultra immunostainer. Finally, sections were rinsed, dehydrated, and coverslipped using a Tissue-Tek Film coverslipper (Sakura, Alphen aan den Rijn, The Netherlands).

### Immunofluorescent Staining for Neurofilament

Sections for immunofluorescent staining were bleached in Autofluorescence Eliminator Reagent (Millipore) according to the manufacturer's guidelines. Sections were then pre-incubated with 5% fetal calf serum diluted in phosphate-buffered saline (PBS) containing 0.25% Triton (PBS-T). Sections were incubated overnight with anti-NF-H antibody (clone TA51, 1:50, Millipore). On the following day, sections were rinsed in PBS-T for 10 min and incubated with Alexa-488-conjugated donkey anti-rat (1:500, Invitrogen) for 2 h at room temperature. Finally, sections were rinsed 3 × in PBS, 1 × in TBS, before being mounted in ProLong Gold Antifade Reagent with 4′,6-diamidino-2′-phenylindole dihydrochloride (DAPI) (Thermo Fisher Scientific).

### Statistics

SSS and mRS were treated as continuous numeric variables, and controls were assumed to score 0 on mRS and 58 on SSS. Correlations between mRS or SSS and NF-L, S100B, or IL-6 were tested using the non-parametric Spearman's rank correlation test to account for non-linear relationships between covariates. Group differences were tested using a Kruskal–Wallis test followed by Dunn's multiple comparison test and multiple linear regression with ischemic stroke and TIA as dummy variables and controls as the base level. NF-L levels were modeled using multiple linear regression with NF-L concentration as the outcome and group (stroke, TIA, or control), age, sex, and BMI as covariates. Statistical analyses were carried out using R 3.5.1. Data are presented as medians and interquartile ranges [25, 75], and *p* ≤ 0.05 was considered statistically significant. Graphs were generated using the Prism 5 software for Mac (GraphPad).

### Ethics

Ethical approval was granted by the local research ethics committee (The Regional Committees on Health Research Ethics for Southern Denmark: J. No. S-20160152G, J. No. S-20160037, and J. No. S-20080042), and the study was reported to the Data Protection Agency.

## Results

The study cohort consisted of 31 ischemic stroke patients, 9 TIA patients, and 29 healthy controls. Each group was mostly composed of males (56–66%), and median age was 58 years in controls, 69 in ischemic stroke patients, and 71 years in TIA patients (Table 1). The majority of ischemic stroke patients were former or current smokers and had average alcohol consumption. The use of blood-thinning, cholesterol-lowering, and anti-inflammatory medication was higher in ischemic stroke patients and TIA patients than in healthy controls (Table 1).

**Table 1:** Baseline characteristics for healthy controls, ischemic stroke, and transient ischemic attack patients.

	**Healthy controls**	**IS**	**TIA**
*N*	29	31	9
Age (median + IQR)	58 (51;64)	69 (58–77.5)	71 (62–75)
Sex [*N* (%) males]	19 (66%)	20 (65%)	5 (56%)
BMI (median + IQR)	27.5 (26.2;28.1) (5 missing)	26.3 (22.6–28.4)	24.9 (22.8–28.7) (1 missing)
SSS (median + IQR)	–	52 (47–55) (2 missing)	58 (56–58)
Converted NIHSS (median + IQR)		3.3 (1.8–5.7) (2 missing)	0.7 (0.7–4.2)
mRS (median + IQR)	–	1 (1;3) (4 missing)	0(0)
Anti-inflammatory medication (%)	1 (3.5%) (10 missing)	11 (35%)	5 (55.5%)
Blood thinning medication (%)	1 (3.4%) (10 missing)	15 (48.4%)	5 (55.5%)
Cholesterol-lowering medication (%)	2 (6.9%) (9 missing)	5 (16.1%)	2 (22.2%)
Smoking (%)			
Never smoked	10 (34.4%)	4 (13%)	4 (45%)
Smoker	5 (17.2%)	8 (26%)	1 (11%)
Former smoker	5 (17.2%)	16 (51%)	3 (33%)
Unknown	3 (10.4%)	3 (10%)	1 (11%)
Missing	6 (20.7%)	0	0
Alcohol (%)			
None	6 (20.7%)	6 (19%)	2 (22%)
</=limit	12 (41.4%)	18 (58%)	6 (67%)
>limit	2 (6.9%)	6 (19%)	1 (11%)
Unknown	2 (6.9%)	1 (4%)	0
Missing	7 (24.1%)	0	0

*N, number of individuals; IQR, interquartile range*.

Ischemic stroke patients had a lower median SSS score (52; IQR 37–55) than did TIA patients (58; IQR 56–58) (Table 1), indicating more severe symptoms on admission in ischemic stroke patients. This was also reflected in higher converted NIHSS scores in ischemic stroke patients (3.3; IQR 1.8–5.7) compared to TIA patients (0.7; IQR 0.7–4.2).

At 3 months of follow-up, ischemic stroke patients had a higher median mRS score (1; IQR 1–3) than TIA patients (0; 0), indicating greater functional disability in ischemic stroke patients following discharge. None of the ischemic stroke patients or the TIA patients experienced a recurrent ischemic stroke within the 3 month follow-up period. Three stroke patients had passed away, and four ischemic stroke patients were not available for a 3 month follow-up interview.

### NF-L Levels Are Significantly Increased in Ischemic Stroke Patients and Correlate With Stroke Severity and Functional Outcome

**Figure 1:**
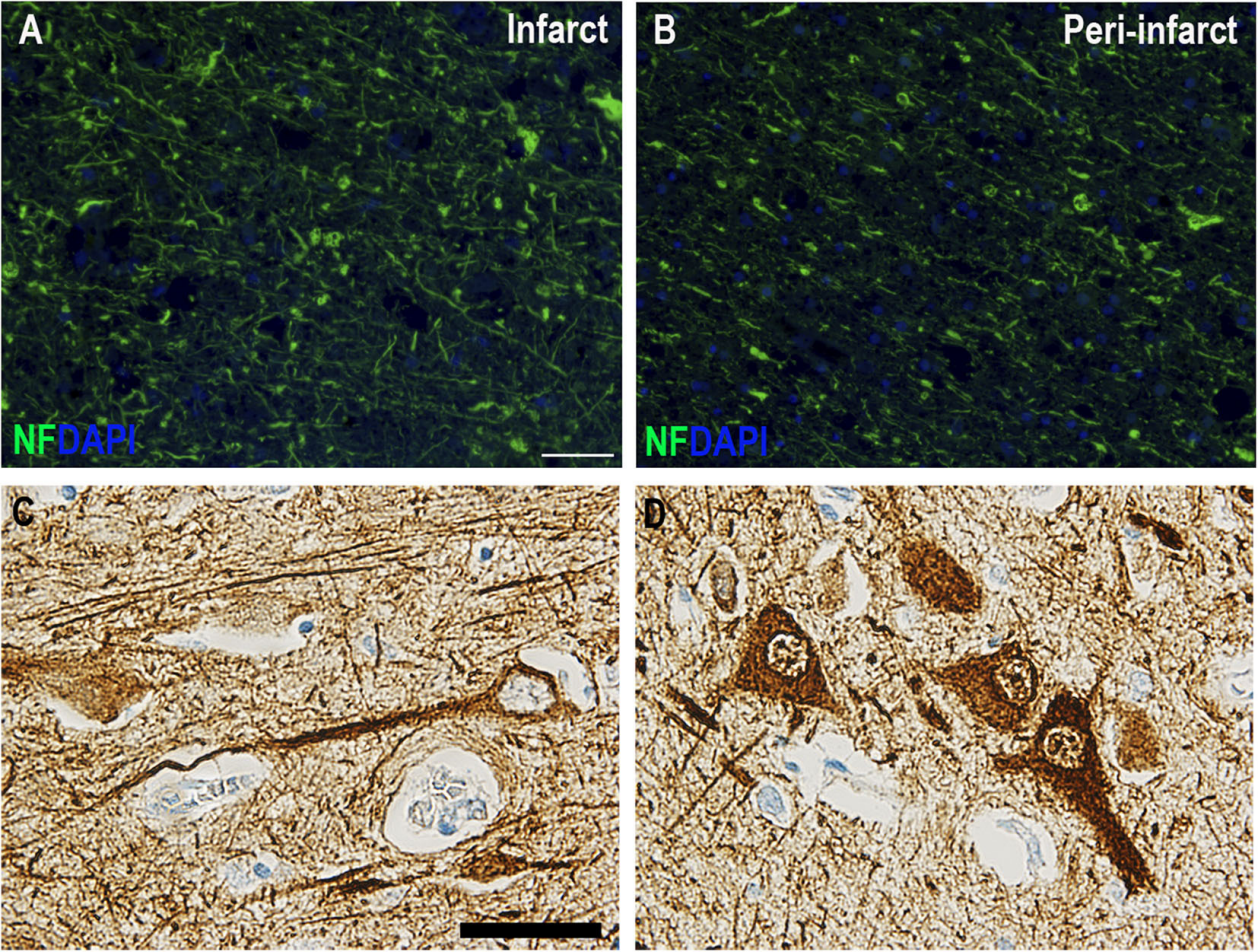
Immunohistochemical staining of neurofilament-positive neurons in postmortem human ischemic brain tissue. **(A,B)** Neurofilament immunofluorescent staining in a >7-day-old right parietal lobe infarct demonstrating increased NF staining in the infarct **(A)** compared to the peri-infarct **(B)**. Scale bar: 100 μm. **(C,D)** High magnifications of neurofilament-positive neurons in paraffin sections from a 3–7-day-old left temporal lobe infarct. Scale bar: 40 μm.

**Figure 2:**
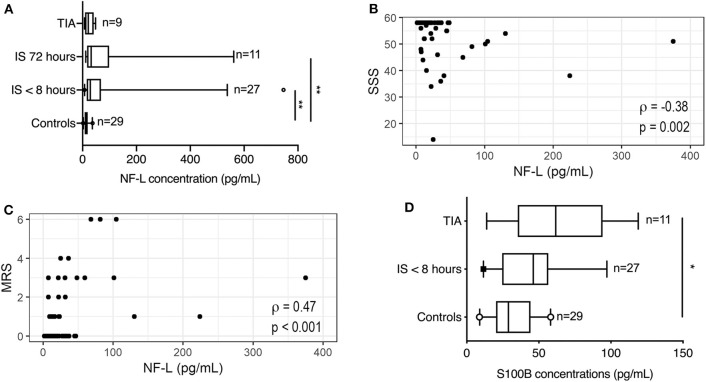
Neurofilament-light chain changes in patients with ischemic stroke. **(A)** NF-L concentrations (pg/mL) increased significantly in the blood of ischemic stroke patients compared to controls (CV = 3.9%). Line: median. Box: interquartile range. Whiskers: 5th–95th percentiles. Kruskal–Wallis test with Dunn's post-hoc test. **(B)** NF-L levels in the blood of patients with ischemic stroke correlated negatively with Scandinavian Stroke Scale (SSS). **(C)** NF-L levels in the blood correlated positively with modified Rankin Scale (mRS) measured 3 months after the ischemic stroke. **(D)** S100B concentrations (pg/mL) increased significantly in the blood of TIA patients compared to controls (CV = 11.5%). Line: median. Box: interquartile range. Whiskers: 5th–95th percentiles. Kruskal–Wallis test with Dunn's post hoc test. *p < 0.05, **p < 0.01. R: Spearman's rho; TIA, transient ischemic attack.

NF immunoreactivity has previously been shown to increase in the ischemic brain following experimental ischemia in rats and mice and the postmortem human ischemic brain (24, 25). In the present study, we also observed intense neurofilament staining in ischemic neurons and in the infarcted brain parenchyma in two postmortem ischemic stroke cases with >7-day-old parietal and 3–7-day-old temporal lobe infarcts (Figure 1). Plasma NF-L levels were significantly increased in ischemic stroke patients acutely (<8 h) (28.70 pg/mL; IQR 15.20–68.30) and at 72 h (31.70 pg/mL; IQR 16.70–98.40) compared to healthy controls (14.10 pg/mL; IQR 7.73–18.96) (Kruskal–Wallis test: chi square = 18.01, p = 0.0004, followed by Dunn's multiple comparisons test: p < 0.01) (Figure 2). In acute ischemic stroke patients, this effect persisted after adjusting for age, sex, and BMI in a multiple linear regression model (ischemic stroke: β = 68.89 pg/mL, SE = 30.05 pg/mL, p = 0.026). NF-L in TIA patients (20.90 pg/mL; IQR 9.30–41.25) did not differ significantly from healthy controls or ischemic stroke patients. NF-L levels in ischemic stroke patients were negatively correlated with SSS (Spearman's rho = −0.38, p = 0.002) and positively correlated with mRS (Spearman's rho = 0.47, p < 0.001), but did not correlate to time after symptom onset (Spearman's rho = −0.07, p = 0.72).

### S100B in Ischemic Stroke Patients Show No Correlation to Functional Outcome

S100B levels were significantly increased in TIA patients (61.73 pg/mL; IQR 35.39–94.25) compared to healthy controls (28.90 pg/mL; IQR 20.28–44.17) (Kruskal–Wallis test: chi square = 6.32, *p* = 0.04, followed by Dunn's multiple comparisons test: *p* < 0.05), whereas S100B levels in ischemic stroke patients (46.10 pg/mL; IQR 24.58–56.52) were comparable to healthy controls (*p* = 0.21). S100B levels did not correlate to mRS (Spearman's rho = 0.21, *p* = 0.17), SSS (Spearman's rho = −0.22, *p* = 0.15), NF-L levels (Spearman's rho = 0.14, *p* = 0.35), IL-6 levels (Spearman's rho = −0.13, *p* = 0.60), or time after symptom onset (Spearman's rho = −0.12, *p* = 0.71).

### IL-6 in Ischemic Stroke Patients Show No Correlation to Functional Outcome

**Figure 3:**
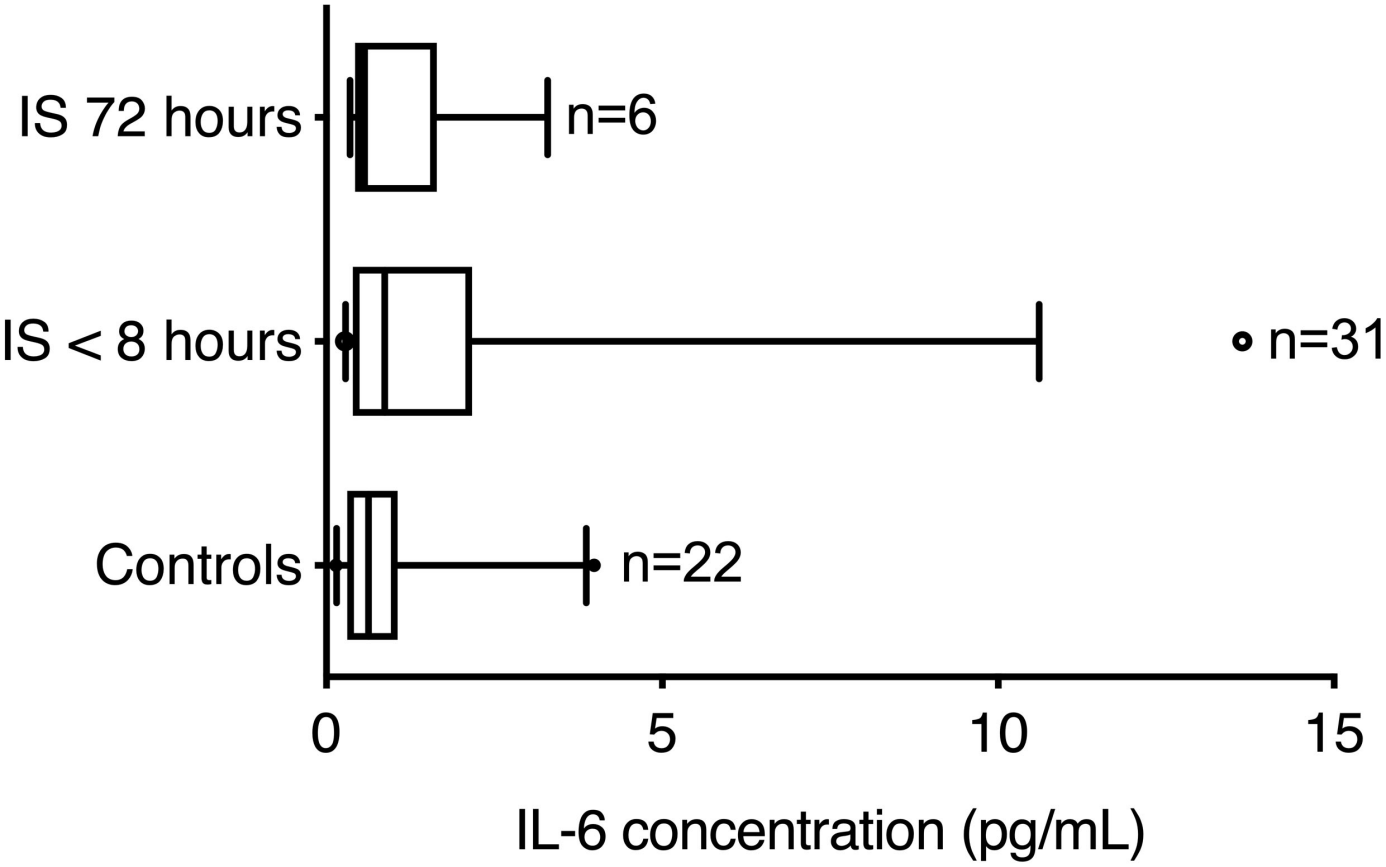
Interleukin-6 levels in patients with ischemic stroke. Blood IL-6 concentrations (pg/mL) were similar in healthy controls and ischemic stroke patients (CV = 4.1%). Line: median. Box: interquartile range. Whiskers: 5th–95th percentiles.

IL-6 levels did not differ significantly between healthy controls (0.63 pg/mL; IQR 0.32–1.06) and ischemic stroke patients, when estimated either within 8 h of symptom onset (0.87 pg/mL; IQR 0.40–2.17) or at 72 h (0.57 pg/mL; IQR 0.43–1.65) (Kruskal–Wallis test: chi square = 2.30, *p* = 0.32) (Figure 3). No correlation was observed between IL-6 and NF-L (Spearman's rho = −0.11, *p* = 0.56), between IL-6 and SSS (Spearman's rho = 0.208, *p* = 0.279), or between IL-6 and mRS (Spearman's rho = −0.239, *p* = 0.211).

### Plasma Cell Adhesion Molecule Concentrations Increase Significantly in Ischemic Stroke

**Figure 4:**
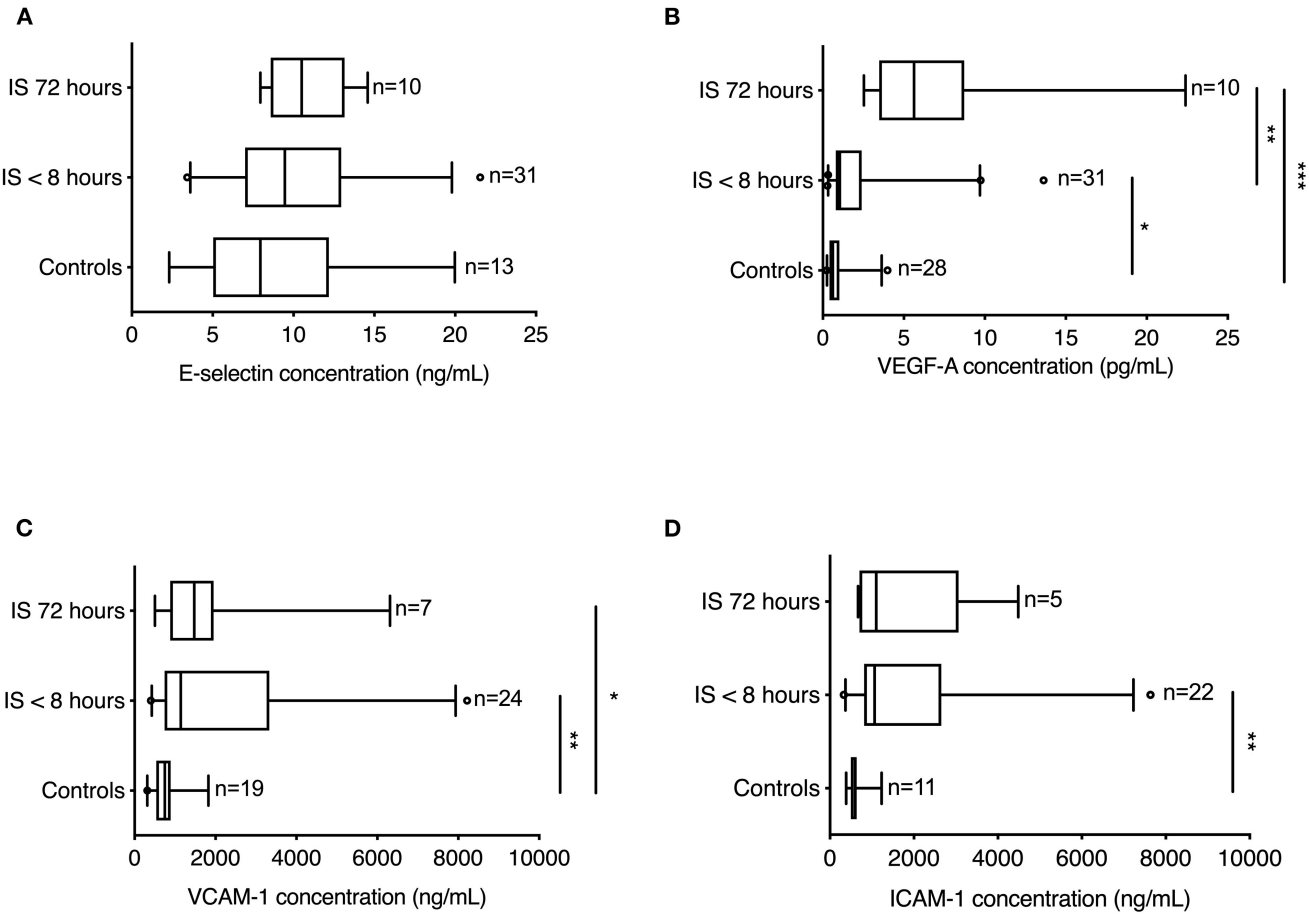
Endothelial and vascular markers in ischemic stroke patients. **(A)** E-selectin concentrations (pg/mL) were similar in healthy controls and ischemic stroke patients (CV = 6.6%). **(B)** VEGF-A concentrations (pg/mL) increased significantly in the first 8 h in ischemic stroke patients compared to healthy controls and showed a further significant increase 72 h after symptom onset (CV = 8.8%). **(C)** VCAM-1 concentrations (pg/mL) were significantly increased both acutely (<8 h) and later (72 h) in ischemic stroke patients compared to healthy controls (CV = 3.6%). **(D)** ICAM-1 concentrations (pg/mL) increased significantly in the first 8 h in ischemic stroke patients compared to healthy controls (CV = 5.8%). Line: median. Box: interquartile range. Whiskers: 5th–95th percentiles. Kruskal–Wallis test with Dunn's post-hoc test. *p < 0.05, **p < 0.01, ***p < 0.001.

We investigated serum concentrations of E-selectin, VEGF-A, VCAM-1, and ICAM-1 in healthy controls and ischemic stroke patients within 8 h of symptom presentation and again at 72 h (Figure 4).

E-selectin levels did not differ significantly between healthy controls (7.95 ng/mL; IQR 5.04–12.19) and ischemic stroke patients, when estimated either <8 h of symptom onset (9.46 ng/mL; IQR 7.01–12.95) or at 72 h (10.50 ng/mL; IQR 8.59–13.16) (Kruskal–Wallis test: chi square = 2.63, *p* = 0.27) (Figure 4A).

Compared to healthy controls (0.61 pg/mL; IQR 0.40–1.01), VEGF-A levels were significantly increased in ischemic stroke patients both <8 h (1.04 pg/mL; IQR 0.80–2.39) (*p* < 0.05) and at 72 h (5.64 pg/mL; IQR 3.48–8.74) (*p* < 0.0001), with a significant increase between 8 and 72 h (*p* < 0.01) (Kruskal–Wallis: chi square = 26.71, *p* < 0.0001, followed by Dunn's multiple comparison test) (Figure 4B).

Compared to healthy controls [749 ng/mL (IQR 536–894)], VCAM-1 levels were found to be significantly increased in ischemic stroke patients within the first 8 h [1,142 ng/mL (IQR 742–3,333)] (*p* < 0.01) and at 72 h [1,476 ng/mL (IQR 880–1,950)] (*p* < 0.05) after symptom onset (Kruskal–Wallis: chi square = 10.80, *p* = 0.0045, followed by Dunn's multiple comparison test) (Figure 4C).

Compared to healthy controls [601 ng/mL (IQR 498–633)], ICAM-1 levels were found to be significantly increased in ischemic stroke patients within the first 8 h [1,065 ng/mL (IQR 814–2,655)] (*p* < 0.01) after symptom onset (Kruskal–Wallis: chi square = 11.98, *p* = 0.0025, followed by Dunn's multiple comparison test) (Figure 4D).

## Discussion

This study identifies plasma NF-L as a predictive marker to separate TIA from ischemic stroke patients in the acute phase (<8 h) after symptom onset. Furthermore, the study adds to the literature identifying NF-L as a reliable biomarker predictive of functional outcome in ischemic stroke patients. We found plasma NF-L levels to be significantly higher in ischemic stroke patients than in healthy controls, whereas NF-L levels in TIA patients were comparable to those in healthy controls. Acute plasma NF-L levels in ischemic stroke patients correlated with stroke severity (SSS score) on admission and with functional outcome (mRS score) 3 months post-stroke. IL-6 and S100B did not show the same correlations. The levels of VEGF-A, ICAM-1, and VCAM-1 levels increased acutely (<8 h) in the blood of ischemic stroke patients compared to healthy controls, with VEGF-A and VCAM-1 levels also increased at 72 h. We did not observe any change in E-selectin levels.

NFs hold great promise as biomarkers for assessing disease activity, monitoring treatment responses, and determining prognosis as their levels in both CSF and blood rise upon neuro-axonal damage and indicate neuro-axonal injury independent of causal pathways. In the present study, we observed increased NF immunoreactivity in the ischemia-affected area compared to normal-appearing tissue in a 3–7-day-old temporal lobe infarct and a >7-day-old right parietal lobe infarct. The increased NF immunoreactivity is possibly related to an increase in detectable NF degradation products in the ischemic core due to structural cytoskeletal alterations in neurons following ischemia (25). Previous studies on postmortem tissues from victims of stroke and other focal lesions using immunostaining with antibodies specific for phosphorylated NF-H have demonstrated that NF phosphorylation (normally confined to axons) occurs aberrantly in perikaria and dendrites of neurons with axons projecting to damaged brain areas (26). This is in line with the present study, where we also used an antibody against NF-H to detect NFs in the ischemic brain. In a case of a 3 week-old human postmortem brainstem stroke (25), NF-H immunoreactivity appeared less pronounced, whereas NF-L immunoreactivity increased within the infarct whereas NF-M and alpha-internexin decreased. NF-L immunoreactivity was also increased in human postmortem brain tissue from a patient with a right middle cerebral artery infarct (24), although the age of the infarct was not reported. The reason for the discrepancy in our findings and the findings by Mages et al. (25) on NF-H immunoreactivity may lie in the differences in the antibodies employed, the age of the infarct, or the anatomical location of the infarct. Mages et al. (25) used a mouse anti-NF-H IgG (clone NE14) that reacts with the 200-kDa NF, whereas we used a rat anti-NF-H IgG (clone TA51) that reacts with the phosphorylated H-chain of the 200-kDa NF. Furthermore, the tissue used by Mages et al. was derived from a 3 week-old brainstem infarct, whereas we used tissue from a 3 to 7-day-old temporal lobe infarct and a >7-day-old parietal lobe infarct, and it is possible that either the anatomical distribution of NFs is different or that by 3 weeks, NF-H immunoreactivity is lost within the infarct core.

Brain proteins can access the blood flow either via CSF drainage through the venous sinuses or by diffusion through the BBB. As ischemic stroke leads to an acute breakdown of the BBB (27), brain proteins quickly leak into the blood. The lack of oxygen and nutrients that accompanies ischemic stroke leads to disruption of the axonal membrane and thus the release of NFs into the interstitial fluid and eventually into the CSF and blood. Patients with ischemic stroke and TIA show a high rate of recurrent ischemic lesions on MRI, particularly within the first months after stroke (28–30). We and others hypothesized, therefore, that blood NF levels could predict neurological outcome after TIA and ischemic stroke.

Serum levels of phosphorylated NF-H increase in ischemic stroke patients 1–3 weeks after symptom onset compared to healthy controls and correlate significantly at 3–6 weeks with baseline NIHSS scores, 1 week mRS scores, and infarct volumes at 6 months (31, 32). However, as the clinical significant correlation between blood NF-H levels and functional outcome does not appear until 1 week after symptom onset, NF-H appears not to be a good biomarker in the acute setting and therefore not a good biomarker to discern between TIA and ischemic stroke.

In the present study, we found that plasma NF-L levels increased acutely in ischemic stroke patients compared to healthy controls, which was not the case for TIA patients. Further, acute plasma NF-L levels in ischemic stroke patients correlated with SSS on admission and predicted functional outcome 3 months post-stroke, independent of age, BMI, and gender. Our findings are in line with a study by De Marchis et al. (13), who found that serum NF-L levels within 24 h of symptom onset were significantly higher in ischemic stroke compared to TIA patients and healthy controls, also after adjusting for age and NIHSS score. However, following adjustment, the authors did not find a correlation between serum NF-L and infarct size on admission or mRS at 3 months. Tiedt et al. (12) found NF-L levels to be increased in ischemic stroke patients at admission (<24 h), days 2, 3, and 7, and at 3 and 6 months post-stroke, with peak levels at day 7. NF-L levels at day 7 correlated with infarct volumes assessed by MRI and with mRS 3 months post-stroke, but after adjustment for age and hypertension, only NF-L levels obtained on days 3 and 7 correlated with infarct volume. Traenka et al. (33) investigated serum NF-L levels within 30-days of symptom onset in ischemic stroke and TIA patients and found significantly higher serum NF-L levels in ischemic stroke patients that were associated with unfavorable outcome at 3 months. This association lost significance after adjustment for NIHSS, however, supporting the potential of serum NF-L as a predictive biomarker in the acute phase.

It is possible that NF-L levels in our patients were influenced by unrecognized neurodegenerative disease that may have contributed to disability. Also, NF-L might have captured aspects of stroke-related tissue injury that are particularly relevant for functional outcome. Conceivably, its specificity for neuronal damage might distinguish plasma NF-L from other blood-based biomarkers with predictive value for stroke outcome reflecting other biological processes, such as copeptin (34), brain natriuretic peptide (35), C-reactive protein (36), astroglial markers GFAP, CHI3L1, and S100B (37), oligodendrocyte marker MBP (37), and IL-6 (14). Whether patients with high plasma NF-L levels on admission benefit from more intensive rehabilitative treatment is beyond the scope of this study.

Astrocytes change from their normal quiescent state into reactive astrocytes whenever damage is inflicted on the CNS. This process of reactive gliosis is characterized by a pronounced increase in GFAP and S100B, supporting their potential role as prognostic markers in ischemic stroke. Serum S100B values obtained in acute ischemic stroke patients 24–72 h after symptom onset, but not at earlier time points, have been shown to predict functional outcome and infarct volume in ischemic stroke patients (38, 39). This, combined with the findings in the present study of no change in S100B levels in acute ischemic stroke patients compared to TIA patients and controls, suggests that S100B is not a valuable marker for diagnosing acute ischemic stroke. The correlation between 24 and 72 h of serum S100B levels and infarct volume and functional outcome, however, suggest that S100B can be used to identify patients at increased risk of specific early neurological complications and as a surrogate marker of cerebral damage and functional outcome.

Elevated IL-6 levels in the CSF of acute stroke patients have been shown to correlate with infarct volume and functional outcome (40, 41), while plasma IL-6 levels obtained within the first week of stroke debut correlated with brain infarct volume, stroke severity and long-term outcome (14, 42–44). Conversely, in line with the present study, other studies have reported no association between serum IL-6 levels and infarct volume or stroke severity (40, 45). As IL-6 levels were investigated in the acute phase (<8 h) post-stroke in the present study and IL-6 is known to peak after 24 h (43), this suggests that IL-6 is not a reliable biomarker in the acute phase after stroke debut.

A recent MRI study demonstrated that the BBB permeability in ischemic stroke patients may be most elevated 6–48 h after stroke debut (27). In support of this, we observed significantly increased plasma levels of VEGF both acutely (<8 h) and 72 h after symptom onset in ischemic stroke patients compared to controls. These findings are in line with previous studies (46–48). Despite the role of VEGF in neuronal survival and angiogenesis (49) and neurovascular remodeling post-stroke (50), VEGF also induces endothelial proliferation and increases endothelial permeability, leading to BBB breakdown (51, 52).

Endothelial markers VCAM-1, ICAM-1, and E-selectin are involved in the adhesion of inflammatory cells to the endothelium and transportation across the BBB to the ischemic area [reviewed in (53)]. Adhesion molecules, such as ICAM-1, VCAM-1, and E-selectin, are increased in endothelial cells as a consequence of ischemic stroke and can be released into the blood (54). Blood levels of ICAM-1, VCAM-1, and E-selectin were higher in ischemic stroke patients than in TIA patients, without a correlation of infarct volume with functional outcome (55). This is in line with our findings demonstrating significantly increased plasma ICAM-1 and VCAM-1 levels in ischemic stroke patients compared to controls although we observed no change in E-selectin. ICAM-1 peaked at 24 h and VCAM-1 at 5-days after symptom onset (55), which is in line with our findings of reduced ICAM-1 levels and persistently increased VCAM-1 levels at 72 h. Our finding of no change in E-selectin was surprising as E-selectin not only is a predictor of ischemic stroke (56) but also increases in the acute phase after ischemic stroke (55, 57–59) and can predict functional outcome (55). It is possible that our sample size was simply too small, suggesting that E-selectin may not be as reliable a biomarker as NF-L.

The strengths of this study are the short median time interval between ischemic stroke debut and blood sampling (<8 h), the sampling of blood prior to thrombolysis, the discrimination of ischemic stroke from TIA patients, and the inclusion of IL-6 and S100B. The limitations are the low number of patients included, the lack of longitudinal sampling (to assess the dynamics of plasma NF-L), and the lack of MRI diagnostics to determine a possible correlation between infarct size and plasma NF-L levels (patients had only CT in the acute phase). Another limitation is that relationships between IL-6 or S100B and SSS or mRS were not age-adjusted. The relationships between NF-L, S100B, or IL-6 and SSS or mRS were tested using Spearman's rank correlation test, a non-parametric test that is better at detecting non-linear relationships. Adjusting for age, sex, and BMI is only possible by assuming a linear relationship between the variables, which could lead to incorrect conclusions regarding the significance of the correlation.

We conclude that our results support the feasibility of quantifying NF-L in acute plasma samples as a potential measure of functional outcome in ischemic stroke patients independently of other prognostic factors such as age, sex, and body mass index. Furthermore, NL-L levels appear to be able to discriminate between TIA and ischemic stroke in the acute phase after symptom onset. Given its correlation with both stroke severity (SSS score) and functional outcome (mRS score), NF-L is a promising biomarker candidate that could be feasibly implemented in a clinical setting. Further studies including more patients, however, should be conducted in order to validate NF-L as a biomarker for acute ischemic stroke.

## Data Availability Statement

The datasets for this article are not publicly available because according to The Danish Data Protection Agency, a general sharing of patient data is not allowed. Requests to access the datasets should be directed to Kate Lykke Lambertsen, klambertsen@health.sdu.dk.

## Ethics Statement

The studies involving human participants were reviewed and approved by The Regional Committees on Health Research Ethics for Southern Denmark. The patients/participants provided their written informed consent to participate in this study.

## Author Contributions

CBS performed NF-L analysis together with JM and helped draft the manuscript. SH recruited all stroke patients with the assistance of AC and CM and all controls together with LF. BC contributed to IL-6 analyses and immunofluorescent detection of neurofilament. MD and RH performed S100B analyses. CS performed statistical analyses. HN and KL conceived the study, interpreted data, and wrote the manuscript. All authors read and approved the manuscript.

## Conflict of Interest

The authors declare that the research was conducted in the absence of any commercial or financial relationships that could be construed as a potential conflict of interest.

## Footnotes

^1^WHO. World Health Organization. Available online at: https://www.who.int.

## Abbreviations

BBB: blood-brain barrier
BMI: body mass index
DAPI: 4′,6-diamidino-2′-phenylindole dihydrochloride
ICAM-1: intercellular adhesion molecule-1
IL-6: interleukin-6
mRS: modified Rankin Scale
NF: neurofilaments
NF-L: neurofilament light chain
NIHSS: National Institute of Stroke Severity Scale
PBS: phosphate-buffered saline
PBS-T: phosphate-buffered saline containing 0.25% Triton
SSS: Scandinavian Stroke Scale
TBS: Tris-buffered saline
TIA: transient ischemic attack
VCAM-1: vascular cell adhesion molecule-1
VEGF-A: vascular endothelial growth factor-A.
